# Enhancing Interaction between Lanthanum Manganese Cobalt Oxide and Carbon Black through Different Approaches for Primary Zn–Air Batteries

**DOI:** 10.3390/ma17102309

**Published:** 2024-05-13

**Authors:** Mario García-Rodríguez, Jhony X. Flores-Lasluisa, Diego Cazorla-Amorós, Emilia Morallón

**Affiliations:** 1 Departamento Química Física e Instituto Universitario de Materiales, Universidad de Alicante, Ap. 99, E-03080 Alicante, Spain; mario.garcia@ua.es (M.G.-R.);; 2 Departamento Química Inorgánica e Instituto Universitario de Materiales, Universidad de Alicante, Ap. 99, E-03080 Alicante, Spain; cazorla@ua.es

**Keywords:** perovskite, carbon material, composites, ORR, synergistic effect, strong interaction

## Abstract

Due to the need for decarbonization in energy generation, it is necessary to develop electrocatalysts for the oxygen reduction reaction (ORR), a key process in energy generation systems such as fuel cells and metal–air batteries. Perovskite–carbon material composites have emerged as active and stable electrocatalysts for the ORR, and the interaction between both components is a crucial aspect for electrocatalytic activity. This work explores different mixing methods for composite preparation, including mortar mixing, ball milling, and hydrothermal and thermal treatments. Hydrothermal treatment combined with ball milling resulted in the most favorable electrocatalytic performance, promoting intimate and extensive contact between the perovskite and carbon material and improving electrocatalytic activity. Employing X-ray photoelectron spectroscopy (XPS), an increase in the number of M-O-C species was observed, indicating enhanced interaction between the perovskite and the carbon material due to the adopted mixing methods. This finding was further corroborated by temperature-programmed reduction (TPR) and temperature-programmed desorption (TPD) techniques. Interestingly, the ball milling method results in similar performance to the hydrothermal method in the zinc–air battery and, thus, is preferable because of the ease and straightforward scalability of the preparation process.

## 1. Introduction

Climate change is a major concern today due to the high levels of pollution from fossil fuels. To address this challenge, society needs to transition towards a renewable and sustainable energy model [1,2]. Therefore, developing systems to store and convert energy from renewable sources is crucial. Electrochemical devices including fuel cells and primary metal–air batteries (ZABs) are potential candidates to meet this purpose [3,4]. ZABs have received intensive research and development activity because of their high theoretical energy density (1086 Wh/kg), abundant reserves of Zn metal, good rechargeability, low manufacturing cost, high safety, and eco-friendliness [5,6]. An additional key advantage of ZABs is their compatibility with other energy storage systems. This allows them to leverage complementary technologies, combining the strengths of each system for a more robust and efficient energy storage solution. This makes ZABs highly attractive for commercialization, and they have garnered significant attention from both academia and industry. The past decade has witnessed a surge in ZAB research, as evidenced by the rapidly increasing number of publications [7].

The main limitation for large-scale implementation in primary ZABs is the slow kinetics of the oxygen reduction reaction (ORR) [8,9,10]. While noble metal catalysts like Pt/C demonstrate excellent ORR activity, their high cost and scarcity render them unsuitable for large-scale applications. Therefore, research is needed to develop cost-effective electrocatalysts for the ORR. The family of metal oxide perovskites of the AMO_3_ type, where A is a rare earth or alkaline earth cation and M is a transition metal cation, is an attractive candidate for this reaction [11,12]. However, limited conductivity hinders their electrocatalytic activity. A solution involves adding a second phase with high electronic conductivity, such as carbon materials. This results in a composite that exhibits significantly enhanced ORR activity compared to the pure perovskite-type metal oxide or carbon material alone. This improvement is attributed to a synergistic effect, where the combined phases work together to improve overall performance [13].

Three possible origins could explain the synergistic effect [14,15]. First, the ligand (electron) effect involves the electronic interaction (charge transfer) between two elements. It is widely used in alloys and metal/metal oxide composites for electrocatalysis and heterogeneous catalysis and is the main reason for improving catalytic performance. Thus, the ligand effect at the interface between the carbon material and the perovskite can change the electronic structure and promote electron transfer. Second, the formation of interfacial heterostructures (i.e., covalent bonds or a new phase at the interface), usually represented as M-O-C interactions [16,17,18], can be at the origin of the synergistic effect for enhanced ORR performance in the strongly coupled hybrids. The M-O-C species are believed to favor the adsorption and desorption of oxygen-containing species from active sites, thereby enhancing the overall reaction [17]. Other authors also relate the improvement in electrocatalytic activity to the intrinsic properties of carbon materials, such as their hydrophobicity, low degree of structural order, surface area, and homogeneity [19,20].

On the other hand, a change in the mechanism by which the ORR takes place in perovskite–carbon composites is also proposed [21]. One possible explanation is that carbon material and perovskite-type metal oxide catalyze different reactions, resulting in a series of 2e^−^ (carbon material) + 2e^−^ (perovskite) reactions. The 2e^−^ reaction of oxygen to hydroperoxide is first catalyzed by carbon material. The hydroperoxide is then desorbed and subsequently adsorbed onto the metal oxide perovskite [22,23]. Then, the perovskite catalyzes the electrochemical reduction of hydroperoxide to hydroxide according to the well-known ORR–perovskite mechanism [24]. While this mechanism appears to be a pseudo-4e^−^ pathway, it does not necessarily exclude the possibility of a direct 4e^−^ reaction occurring simultaneously. Furthermore, there is no evidence that this two-step two-electron mechanism can be applied to all perovskite composites.

There is a wide variety of carbon materials that can be combined with perovskite-type metal oxides, which have demonstrated an improvement in electrocatalytic activity. Carbon black (CB) materials are the most commonly used because they increase the electrical conductivity and even exhibit some inherent ORR activity (some examples are Vulcan XC-72(R) [25,26,27], acetylene black [28,29], Ketjen black [30], and Super P [31,32]). Several studies have explored incorporating carbon materials with even higher electrical conductivity, such as carbon nanotubes [33,34] and graphene-based materials [35,36], or higher surface areas like activated carbons [37]. Alternatively, some research has focused on nitrogen-doped carbon materials, which possess inherent active sites for the ORR, further enhancing the activity of the metal oxides [38,39].

The way of mixing perovskite-type metal oxides with carbon materials is also a critical aspect affecting the electrocatalytic activity [40]. The most commonly used method involves physical mixing after the synthesis of the metal oxide perovskite [41,42,43], either using a mortar or specific equipment such as a ball mill. Another approach involves chemical synthesis in the presence of the carbon material. This method aims to create a stronger interaction between the perovskite and the carbon material. The literature explores various chemical synthesis techniques, including the hydrothermal method [39,44,45], electrospinning [46], and chemical vapor deposition (CVD) [47,48]. Some works simply ultrasonicated perovskite and carbon to make the mixture [49], but other works such as the one published by Wang et al. [50] developed a perovskite–carbon Super P composite through ball milling both components for ZAB applications. In addition, hydrothermal treatment has been used for developing a LaNiO_3_-nanorod/graphene composite for ZAB applications [44]. However, the potential benefits of combining physical and chemical treatments when mixing the two components post-perovskite synthesis have not yet been studied.

This work investigates the electrocatalytic activity of La_0.6_Mn_0.7_Co_0.3_O_3_ perovskite for the ORR by incorporating carbon materials through various mixing methods. Prior research has identified LaMn_0.7_Co_0.3_O_3_ as the most electroactive composition in the LaMn_1−x_Co_x_O_3_ series due to the formation of oxygen vacancies and an enhanced surface concentration of active M-site cations [27]. These vacancies act through a charge compensation mechanism, improving overall performance. Additionally, studies on La_1−x_MnO_3_ oxides suggest that creating A-site vacancies can further increase the concentration of active sites on the surface [51]. Then, the combination of both factors involving M- and A-sites for the ORR in lanthanum manganese cobalt oxides can be interesting in improving the ORR mechanism towards the overall four-electron reaction, and the interaction with carbon material can also be enhanced [52]. Combining these strategies, this work explores the potential benefits of manipulating both M- and A-sites in the La_0.6_Mn_0.7_Co_0.3_O_3_ perovskite for improving ORR activity, particularly towards the favorable four-electron reaction pathway. To achieve this, various mixing methods are employed, including the mortar method, ball milling, and a combination of ball milling with low-temperature hydrothermal and high-temperature thermal treatments. Additionally, the most electroactive samples are evaluated in a primary ZAB.

## 2. Experimental

The materials and reagents used, as well as the characterization techniques, can be found in the Supporting Information.

### Synthesis Procedure

The synthesis of La_0.6_Mn_0.7_Co_0.3_O_3_ (L6M7C3O) was performed by a sol–gel method similar to that described in the literature [53]. The mass of reagents was adjusted to obtain approximately 1.0 g of product, employing a 1:1.5:1 molar ratio for EDTA, citric acid, and the sum of cation precursors, respectively. The cation molar ratio was adjusted to 0.6:0.7:0.3 for La(NO_3_)_3_·6H_2_O, Mn(NO_3_)_2_·xH_2_O, and Co(NO_3_)_2_·6H_2_O, respectively. First, EDTA was dissolved in a mixture of 41 mL of deionized water and 3.3 mL of NH_3_. The stoichiometric amounts of metal precursors and citric acid were added to the EDTA solution. Then, NH_3_ was slowly dropped into the solution to set the pH at 9 to form a stable sol complex. Then, the solution was stirred at 80 °C for 6 h and subsequently dried at 150 °C overnight in a stove to obtain the gel. The gel was heated at 500 °C for 1 h. Finally, this product was ground and calcined at 700 °C for 6 h to form the perovskite metal oxide materials.

The metal oxide–carbon material samples were prepared using different methods, with a 1:1 weight ratio between both components. The mortared sample was obtained by mixing both components (metal oxide and carbon black) employing an agate mortar for 15 min (L6M7C3O_Mortar). The ball-milled sample (L6M7C3O_BM) was prepared by mixing both components using a planetary mill Retsch PM 200 under 350 rpm for 30 min, following the milling, using the optimal milling conditions reported in our previous work [40]. The hydrothermal sample (L6M7C3O_HT) was obtained by sonicating 250 mg of the L6M7C3O_BM sample with 28 mL of H_2_O and 2 mL of NH_3_ for 30 min. Then, the dispersion was transferred to a Teflon reactor and introduced into an autoclave which was kept for 15 h at 150 °C. Finally, the sample was washed until a neutral pH was reached and then dried at 80 °C overnight. The thermally treated sample (L6M7C3O_TT) was obtained by heating 250 mg of the L6M7C3O_BM sample up to 500 °C for 3 h at a heating rate of 5 °C/min under an N_2_ atmosphere.

## 3. Results and Discussion

### 3.1. Bulk Structure and Composition

In order to investigate the potential impact of the mixing procedure on the electrocatalytic activity, the materials were characterized by TEM microscopy to observe any changes in the perovskite nanoparticles resulting from the mixing procedure. These changes could affect the electrochemical activity. Figure S1 depicts a TEM image of L6M7C3O perovskite, revealing agglomerates of irregularly shaped nanoparticles of around 25 nm. Figure S2 displays the TEM images of the perovskite–carbon composites produced using different methods. The samples exhibit irregular agglomerates of spherical shape, with the perovskite particles retaining their size and shape. The size of the agglomerates varies between samples, with L6M7C3O_Mortar and L6M7C3O_BM samples exhibiting a similar mean diameter of 35 nm, while the mean diameter for the L6M7C3O_HT and L6M7C3O_TT samples is 29 and 57 nm, respectively. These findings demonstrate that hydrothermal treatment reduces the mean diameter of the perovskite–carbon material nanoparticles, while the thermal treatment sinters the nanoparticles.

**Figure 1:**
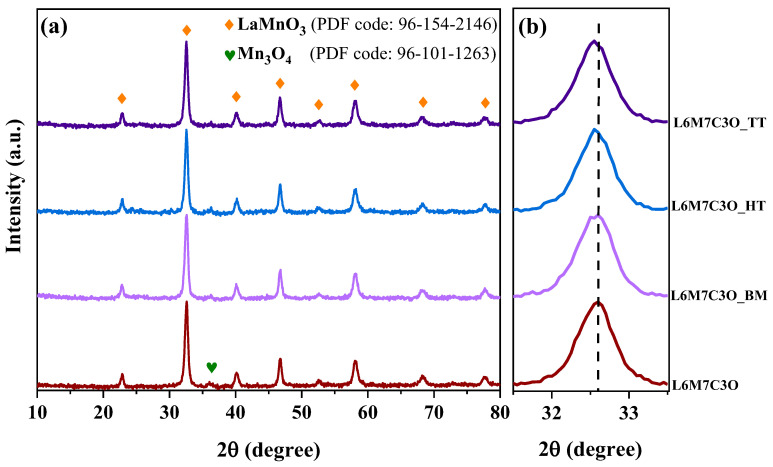
(**a**) X-ray diffraction patterns of the L6M7C3O-based materials and (**b**) zoom on the diffraction line at 32–33°.

For optimal catalytic activity, symmetrical crystal structures in the metal oxide are desired. These configurations facilitate the interaction between the reactants and M site cations, which are the active sites for the ORR. X-ray diffraction (XRD) was employed to characterize the crystalline structure of the metal-oxide–perovskite-type materials. The L6M7C3O_Mortar sample was excluded as mortar mixing is known to not alter the crystal structure [40]. Figure 1 displays the XRD patterns obtained for the perovskite-based materials showing peaks at 2θ = ~23, 33, 40, 47, 58, and 68°, which correspond to the (0 1 0), (0 1 1), (1 1 1), (0 2 0), (1 2 1), and (0 2 2) planes. These peaks can be indexed to a cubic perovskite phase LaMnO_3_ belonging to the Pm-3m space group (PDF code: 96-154-2146). The results confirm the successful incorporation of cobalt into the LaMnO_3_ crystal structure since no other cobalt oxides are detected. A magnification of the XRD signal in the region 32–33° (Figure 1b) reveals a slight shift in the diffraction peak towards lower values of 2θ in the samples mixed with carbon, compared to the pure perovskite (L6M7C3O). Despite this, the lattice parameter of the perovskite remains largely unaffected upon incorporation of the carbon material. On the other hand, the formation of La vacancies in the perovskite promotes the formation of M-cation species, such as manganese oxides [51]. The small peak at around 36° indicates the existence of the Mn_3_O_4_ oxide which has a tetragonal phase and belongs to the I4_1_/amd space group (PDF code: 96-101-1263).

Table 1 presents the crystallographic parameters obtained from the XRD patterns. The Rietveld refinement function of the Highscore Plus software (4.9 version) was employed to accurately estimate the concentration of LaMnO_3_ and Mn_3_O_4_ phases in the samples. Table 1 shows that the percentage of crystalline Mn_3_O_4_ species, which is close to 11% in the pure oxide, increases to 14.7% in the L6M7C3O_HT sample and decreases to 9.8% in the L6M7C3O_TT sample. Hydrothermal treatment preferentially drives the formation of Mn_3_O_4_ species, while thermal treatment favors the formation of purer perovskite phases. The Mn_3_O_4_ phase, previously reported to be active for the ORR [54], could contribute to improve the electrocatalytic activity due to a potential synergistic effect between both metal oxides. The ball-milling treatment did not significantly affect the amount of Mn_3_O_4_ present, which remained around 11.5%—similar to the untreated perovskite (L6M7C3O sample). Next, given the importance of crystallite size in the catalytic performance, it was calculated by applying the Scherrer equation for the peaks at 22–23° and 47–48° [55]. The crystallite size of phases indicates that the LaMnO_3_ structure has particle sizes in the range of 17–19 nm in accordance with TEM images. In contrast, the Mn_3_O_4_ phase exhibits a decrease in particle size when the metal oxide is mixed with carbon black by the different methods.

As the perovskite structure is the main crystallite phase, their lattice parameters were calculated using the following equations [55]:

(1) $$d=\frac{\lambda }{2\sin\theta }$$

(2) $$\frac{1}{d^{2}}=\frac{h^{2}+k^{2}+l^{2}}{a^{2}}$$

where d is the interplanar distance, (hkl) are the Miller indices related to the interplanar distance, and a is the lattice parameter.

### 3.2. Surface Structure and Composition

**Figure 2:**
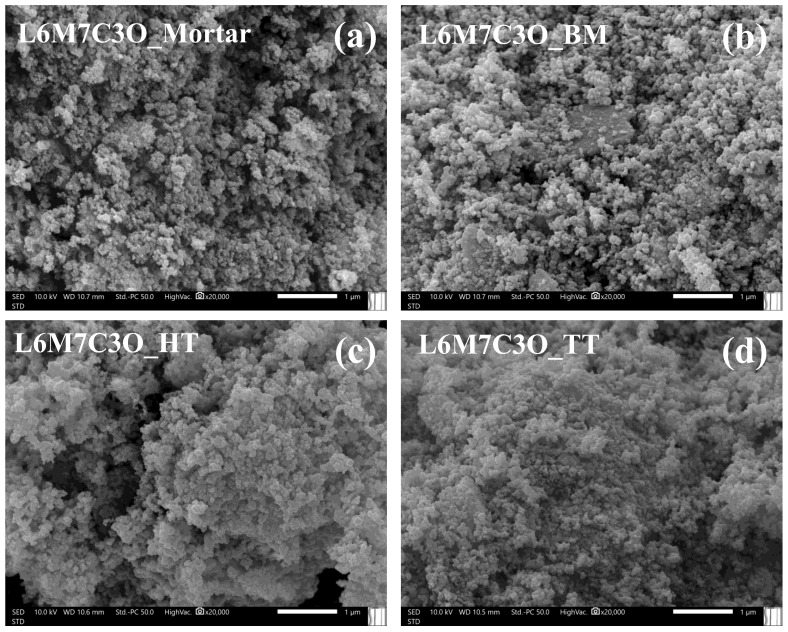
SEM images captured for (**a**) L6M7C3O_Mortar, (**b**) L6M7C3O_BM, (**c**) L6M7C3O_HT, and (**d**) L6M7C3O_TT. Magnification: ×20,000.

Porosity is desirable for electrochemical applications because it provides a larger surface-active area for the reactions, which facilitates the diffusion of reactants and products, ultimately favoring the overall electrocatalytic activity. Therefore, the surface morphology of the metal-oxide-perovskite-based materials was characterized by SEM. At a scale of 5 μm (Figure S3), all samples exhibit spherical nanoparticle agglomerations of similar size, around 10–15 μm in diameter. However, higher magnification reveals significant differences in particle morphology due to the mixing treatment of the components (Figure 2). The L6M7C3O_Mortar and L6M7C3O_BM samples, subjected to mechanical treatment, exhibit greater dispersion, offering a three-dimensional appearance. Conversely, samples subjected to a chemical and thermal treatment (L6M7C3O_HT and L6M7C3O_TT, respectively) display nanoparticle agglomeration, presumably due to a slight degree of sintering caused by the treatment. Therefore, these treatments seem to enhance the contact between the perovskite and Vulcan nanoparticles by causing higher compaction. Moreover, the nanoparticle size for all samples remained unchanged by the mixing treatment.

To assess the potential impact of the mixing method on the porosity of the composites (excluding the mortar-prepared sample), N_2_-adsorption isotherms were obtained at 77 K (Figure S4). The isotherms displayed a type II characteristic according to the IUPAC classification, which is indicative of non-porous materials [56]. Table S1 summarizes the textural properties derived from the N_2_ isotherms. Interestingly, the L6M7C3O_HT sample exhibited the lowest BET surface area (81 m^2^/g), likely as a consequence of the sintering caused by the mixing method.

**Figure 3:**
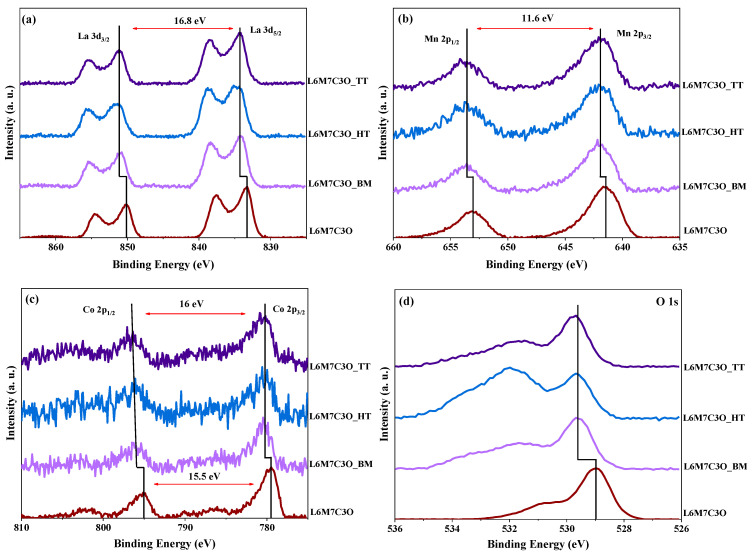
X-ray photoelectron signals obtained from (**a**) La 3d, (**b**) Mn 2p, (**c**) Co 2p, and (**d**) O 1s for the different composites.

Previous studies reported the importance of different M-site cation oxidation states and chemisorbed oxygen species on the surface for the ORR catalytic activity [57]. Thus, XPS is a valuable technique for characterizing the surface of materials and distinguishing Mn and Co oxidation states and different oxygen species formed by the different mixing treatments. Figure 3 depicts the La 3d, Mn 2p, Co 2p, and O 1s core-level spectra for the different materials studied.

The La 3d region (Figure 3a) is characterized by two well-differentiated spin–orbit components at around 834.1 eV (La 3d_5/2_) and 851.0 eV (La 3d_3/2_), showing a multiplet structure [53]. The main difference observed is a binding energy shift towards higher energies in the materials mixed with carbon black. This shift can be attributed to the electronegative influence of the carbon material, which alters the metallic environment through charge withdrawal. This effect, as observed in the following sections for Mn and Co cations, can be related to the ligand effect, which is one of the factors contributing to the synergistic effect observed in these materials [58]. Moreover, the separation of the La 3d_5/2_ and La 3d_3/2_ peaks in all samples corresponds to 16.8 eV, which is related to the La^3+^ species [59]. The Mn 2p spectra (Figure 3b) show two asymmetric spin–orbit signals at around 641.7 and 653.3 eV, which are attributed to Mn 2p_3/2_ and Mn 2p_1/2_, respectively. This asymmetry suggests the presence of different Mn oxidation states, necessitating peak deconvolution for further analysis. The separation between the Mn 2p_3/2_ and Mn 2p_1/2_ peaks of 11.6 eV in all samples indicates the predominance of the Mn^3+^ species [60]. Next, the Co 2p spectra (Figure 3c) display a pair of asymmetric spin–orbit components at 780.6 and 796.1 eV, which can be attributed to Co 2p_3/2_ and Co 2p_1/2_, respectively. In contrast to the La and Mn spectra, these peaks exhibit variations in separation between the samples. According to the literature, a separation of 15 eV is related to Co^3+^ species, while a separation of 16 eV is related to Co^2+^ species [61]. Based on this information, the L6M7C3O sample has a mixture of Co^3+^/Co^2+^ oxidation states. Thus, the hydrothermal and thermal treatments seem to increase the peak separation, suggesting a higher presence of Co^2+^ species. The O 1s spectrum (Figure 3d) shows significant differences, and the presence of the carbon material significantly alters this region. Hence, peak deconvolution is necessary to quantify the contribution of the species in the O 1s, Mn 2p, and Co 2p spectra.

**Figure 4:**
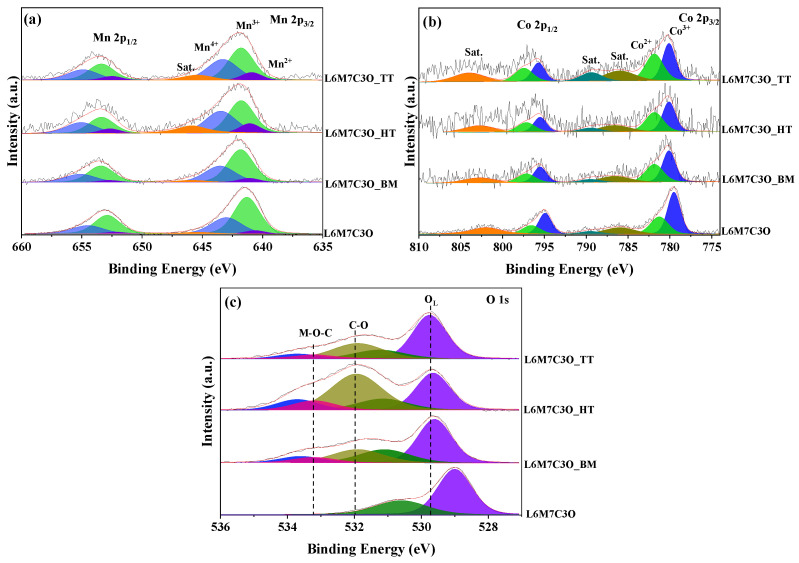
Deconvolution of X-ray photoelectron spectra: (**a**) Mn 2p, (**b**) Co 2p, and (**c**) O 1s.

Figure 4a shows the deconvolution of the Mn 2p_3/2_ spectra, revealing three contributions at 640.5, 641.4, and 643.0 eV. Similarly, the Mn 2p_1/2_ spectra can be deconvoluted into three peaks at 652.0, 641.4, and 643.0 eV, assigned to Mn^2+^, Mn^3+^, and Mn^4+^, respectively [62].

An additional satellite peak at around 645.4 eV is attributed to Mn^2+^ [63]. While Mn^3+^ is the dominant oxidation state across all samples, its relative abundance varies. The L6M7C3O and L6M7C3O_BM samples mainly contain Mn^3+^ and Mn^4+^ species, while the chemical and thermal treatments of L6M7C3O_HT and L6M7C3O_TT samples favor the appearance of Mn^2+^. In addition, the Mn^4+^/Mn^3+^ ratio (Table 2) is higher in the L6M7C3O_HT and L6M7C3O_TT samples, suggesting that the formation of Mn^2+^ occurs at the expense of Mn^3+^ species. 

Furthermore, the higher presence of Mn^4+^ is expected to improve the interaction between M-site cation and oxygen species [64,65]. Additionally, its higher oxidation ability can accelerate the chemical disproportionation of HO_2_^−^ species [64], thereby enhancing the electrocatalytic activity of the ORR.

Figure 4b shows the deconvoluted Co 2p spectra. The Co 2p_3/2_ and Co 2p_1/2_ signals are divided into two peaks assigned to Co^3+^ (at 779.8 and 794.8 eV) and Co^2+^ (at 781.2 and 796.2 eV) [66]. Table 2 shows variations in the Co^3+^/Co^2+^ ratio, with an enrichment of Co^2+^ compared to Co^3+^ in the L6M7C3O_HT and L6M7C3O_TT samples. This effect results from the increase in Mn^4+^ species on the surface, which provokes the reduction of Co^3+^ species to Co^2+^ to keep the electroneutrality on the metal surface.

The deconvolution of the O 1s spectra (Figure 4c) reveals five contributions: two assigned to the metal oxide perovskite, two assigned to the carbon material, and the fifth attributed to the interaction between both, represented by the M-O-C interaction. The peaks at 529.0 and 531.0 eV correspond to lattice oxygen species (O^2−^) and adsorbed oxygen species (O^−^, O^2−^, or O_2_^2−^) of the perovskite, respectively. The peaks at 532.0 and 534.0 eV are associated with the C-O and C=O species of the carbon material, respectively [67]. Finally, the peak at around 533.0 eV is associated with the M-O-C species [27,68]. Additionally, differences are observed in the contribution of the C-O and M-O-C species. The hydrothermal treatment in the L6M7C3O_HT sample increases the number of C-O species in the carbon material, which potentially contributes to a stronger interaction between the carbon material and the perovskite, giving rise to a higher contribution of the M-O-C interaction [40]. Thus, the ratio between the M-O-C species and the lattice oxygen increases from 0.15 in the L6M7C3O_BM sample to 0.32 in the L6M7C3O_HT sample (Table 2). Moreover, the L6M7C3O_TT sample exhibits a more defined perovskite structure, as evidenced by XRD. This aligns with the lower ratio of oxygen from carbon to lattice oxygen among the studied samples, which can be attributed to the formation of stronger lattice oxygen bonds within the perovskite structure.

### 3.3. Interaction between Metal Oxide Perovskite and Carbon Material

The interaction strength between perovskite and carbon material can be a good descriptor to determine the ORR activity of a composite. Therefore, the further characterization of this interaction beyond the determination of M-O-C interactions using XPS is necessary. In this sense, the temperature-programmed reduction in H_2_ (TPR-H_2_) and temperature-programmed desorption (TPD) techniques were reported to be useful in providing information about the existence of this interaction [27,40,69].

**Figure 5:**
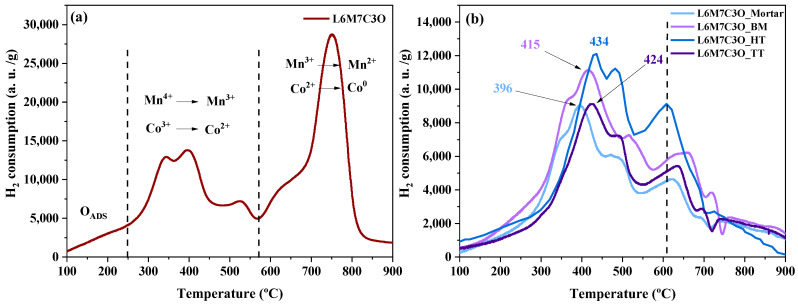
TPR-H_2_ profile of (**a**) LMCO sample and (**b**) perovskite–carbon composites obtained by mortar mixing, ball milling, and hydrothermal and heat treatments.

TPR-H_2_ is a widely used technique in catalysis to study the reducibility of catalytic species, including perovskite-type metal oxides. Significant differences in reduction behavior have been reported for perovskite–carbon composites compared to unmixed metal oxides [27,69]. Carbon material significantly influences the environment and affects the reducibility of the metal oxides due to its inherent reducing characteristic. Figure 5a shows the reduction profile of L6M7C3O perovskite without carbon for comparison purposes.

The region between 150 and 250 °C corresponds to the removal of adsorbed oxygen species. In the region between 250 and 560 °C, the reduction of Mn^4+^ to Mn^3+^ occurs, overlapping with the reduction of Co^3+^ to Co^2+^ [70,71]; these reduction processes take place in two distinct processes that are difficult to assign. The peak at around 525 °C could be ascribed to either the reduction of Co^3+^ to Co^2+^, due to the lower proportion of Co compared to Mn, or the reduction of residual Mn^4+^ and Co^3+^ species that were not reduced at lower temperatures. Finally, a small broader peak at 650 °C related to the reduction of Co^2+^ to Co^0^ and a main peak at around 750 °C associated with the reduction of Mn^3+^ to Mn^2+^ are observed [72]. The large hydrogen consumption is attributed to the Mn^3+^ species already existing in the perovskites and those resulting from the Mn^4+^ reduction. Figure 5b presents the reduction profiles of the perovskite–carbon composites. Clear differences are observed compared to pure perovskite material, namely the disappearance of the major reduction peak at 750 °C and a decrease in hydrogen consumption. The reduction by hydrogen occurs mainly in one peak, ranging between ~400 and ~420 °C, for these samples. This means that in this region, the reduction of Mn^4+^ to Mn^3+^ and Co^3+^ to Co^2+^ occurs (similar to what occurs in the pure metal oxide perovskite). However, the disappearance of the hydrogen consumption at 750 °C indicates that the reduction of Mn^3+^ to Mn^2+^ takes place mainly through the reaction with carbon and at temperatures lower than the reaction with hydrogen.

**Figure 6:**
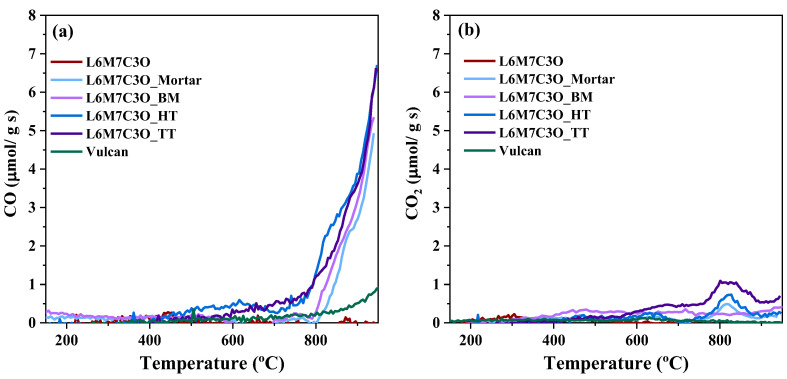
(**a**) CO and (**b**) CO_2_ TPD profiles of perovskite–carbon composites.

The TPD technique is a highly effective analytical tool for investigating the strength and nature of the interfacial interaction in perovskite–carbon composite materials. These experiments provide valuable insights into these interactions, which have been identified as key factors in the electrocatalytic activity of the composite [14]. Figure 6 presents the TPD profiles for the different materials in which CO and CO_2_ were the main desorbed species identified.

The absence of O_2_ desorption in the TPD experiment confirms the thermal stability of the perovskite component under the experimental temperature range, which does not result in dioxygen evolution. Moreover, the desorption of CO and CO_2_ from the pure perovskite metal oxide is negligible. In the case of Vulcan, a small desorption of CO at high temperature is observed. A quantitative determination of the desorbed species together with the experimental and theoretical oxygen amounts is presented in Table 3.

Figure 6 shows that CO is the dominant desorbed species, with some CO_2_ desorption also observed at high temperatures. The desorption of CO and CO_2_ is a result of the carbothermal reduction reaction between the metal oxide perovskite and the carbon material, which occurs due to their intimate interaction at the interface [40]. It is expected that a stronger and more extensive interaction, represented by the M-O-C species, will result in higher desorption at lower temperatures. The different treatments performed on the perovskite–carbon composites result in variations in the TPD profiles of the samples (Figure 6a). Notably, the L6M7C3O and Vulcan samples separately desorb significantly lower amounts compared to the composites, indicating that the interaction between components is crucial in the observed desorption profiles. Furthermore, the total amount of desorbed oxygen due to the reaction between the carbon material and the perovskite can be calculated (Table 3). The table also includes the maximum theoretical value that corresponds to the total amount of oxygen present in the perovskite. Equation (S5) shows how this theoretical maximum value has been calculated. The results show that the highest desorption occurs in samples L6M7C3O_HT and L6M7C3O_TT, followed by L6M7C3O_Mortar and L6M7C3O_BM samples. In terms of the temperature at which CO desorption begins, it starts at around 770 °C in L6M7C3O_HT and L6M7C3O_TT samples, while in the L6M7C3O_Mortar and L6M7C3O_BM samples, it begins at around 800 °C. This demonstrates a stronger and more extensive degree of interaction between perovskite and Vulcan in the L6M7C3O_HT and L6M7C3O_TT samples, highlighting the positive effect of the hydrothermal treatment in improving the interaction of both components.

### 3.4. Electrochemical Characterization

**Figure 7:**
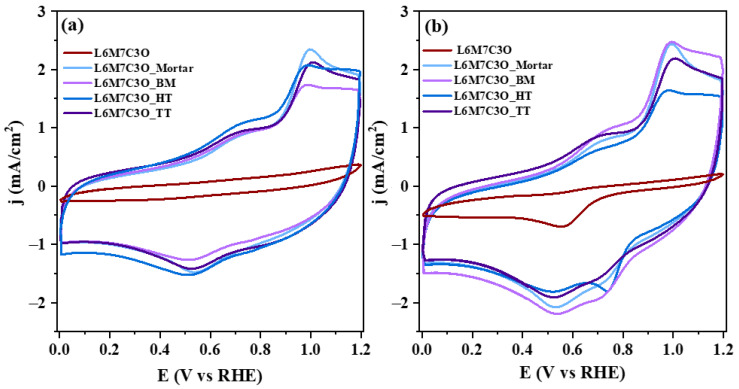
Cyclic voltammograms for the different perovskite–carbon composites in 0.1 M KOH solution saturated with either (**a**) N_2_ or (**b**) O_2_. Scan rate: 50 mV/s.

The different physicochemical and morphological properties amongst the samples could suggest a different electrocatalytic activity. Cyclic voltammetry tests were performed in a N_2_-saturated 0.1 M KOH solution and the results are depicted in Figure 7a.

The main difference observed is the lower electrochemical capacitance and conductivity of the pure metal oxide perovskite sample compared to the perovskite–carbon composites. This is evidenced by the tilted voltammogram of the pure perovskite, which often indicates a high ohmic resistance that overlaps with the recorded current densities. The perovskite–carbon composites display two faradaic processes related to the Mn^2+^/Mn^3+^ redox process occurring at around 0.7 V and a peak around 1.0 V related to the formation of MnOOH species [73,74]. No significant differences are observed in the voltammograms of the composites.

**Figure 8:**
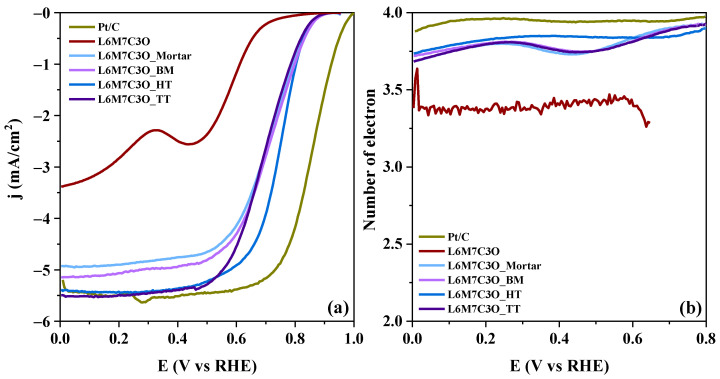
(**a**) Linear sweep voltammograms for the different composites in 0.1 M KOH solution saturated with O_2_ at 1600 rpm, v = 5 mV/s; (**b**) number of electrons transferred in ORR using the current measured at the ring electrode.

As explained in the introduction, combining cations at the M-site and A-site can be beneficial in increasing the surface concentration of active sites in metal oxide perovskites. For this reason, Table 4 compares the ORR parameters of the L6M7C3O_Mortar material prepared in this study with samples with different compositions previously published and prepared using a similar mortar-based approach (LaMn_0.7_Co_0.3_O_3__Mortar [27] and La_0.6_MnO_z__Mortar [51]). Figure S5 compares the LSV obtained during the ORR for all samples mixed with carbon material using mortar (L6M7C3O_Mortar, LaMn_0.7_Co_0.3_O_3__Mortar, and La_0.6_MnO_z__Mortar). The results demonstrate that the L6M7C3O_Mortar prepared in this study exhibits better electrocatalytic behavior, evidenced by its lower Tafel slope and higher E_1/2_ value. The enhanced electrocatalytic activity of the L6M7C3O_Mortar sample is then a consequence of the optimized composition of La and Co species. For this reason, this specific composition for the metal oxide perovskite is the one that has been used in the different preparation methods described in this study. The observed improvement with this specific composition can be related to some combined effects, such as the generation of charge compensation mechanisms due to differences in the oxidation states of the cations; the occurrence of synergy between the two cations; the induction of altered (and more favorable) arrangements in the resulting composite, like porous structures that facilitate the diffusion of electrolytes or oxygen into the perovskite material; or the formation of additional metal redox pairs [53,75]. Figure 8 shows the electrochemical activity towards the ORR evaluated in an RRDE in a 0.1 M KOH electrolyte for the samples in which the carbon material has been introduced through different approaches.

Table 4 displays the main electrochemical parameters of the ORR. The L6M7C3O sample presents an E_onset_ of 0.76 V at −0.10 mA/cm^2^, showing the least favorable result in the series.

It can be observed that the electrocatalytic activity improves for the samples with carbon material. Moreover, the results demonstrate a similar performance among the samples mixed with carbon material, although differences are detected in terms of the half-wave potential and limiting current density. The half-wave potential (E_1/2_) is a widely accepted indicator for evaluating ORR performance, and higher values of E_1/2_ correspond to a lower overpotential to the reaction [76]. Thus, the sample that shows the higher ORR activity is the L6M7C3O_HT sample with the highest E_1/2_ value of 0.75 V. Concerning the limiting current density (j_lim_), the samples subjected to chemical treatment (L6M7C3O_HT and L6M7C3O_TT) are the ones that present the largest current density, −5.36 and −5.40 mA/cm^2^ at 0.4 V, respectively.

The Tafel analysis is an effective tool for studying electron rate-determining processes. In general, small Tafel values imply better ORR activities [77]. As shown in Table 4 and Figure S6, the Tafel slopes for the samples decrease when carbon material is present. In all cases, the results are close to 60 mV/dec, showing that the carbon material influences the electron transfer kinetics.

The number of electrons transferred in the ORR can be observed in Figure 8b. All perovskite–carbon composites exhibit a nearly four-electron transfer pathway, with the L6M7C3O_HT sample showing the highest value of electrons transferred at 0.4 V. However, the limiting current obtained for this sample shows that this electrocatalyst can be an alternative to commercial Pt/C for this reaction.

Then, the superior electrocatalytic activity observed in the L6M7C3O_HT sample compared to the others can be attributed to its physicochemical and morphological properties. First, a higher presence of the electroactive compound Mn_3_O_4_ was detected in the L6M7C3O_HT sample, which likely contributes to an improvement in electrocatalytic activity. Second, the extent of interaction between the perovskite and the carbon material is among the highest. Moreover, the positive synergistic effect between both metal oxides cannot be ruled out.

XPS analysis revealed a higher presence of the M-O-C species in the L6M7C3O_HT sample, indicating the strongest interfacial interaction. Additionally, TPD showed the highest amount of desorbed oxygen for the L6M7C3O_HT sample, further demonstrating a more intimate and extensive contact between the components. The L6M7C3O_TT sample also showed a similar amount of oxygen desorbed, highlighting the positive influence of both hydrothermal and thermal treatments in improving the interaction between perovskite and carbon material. On the other hand, surface morphology studies have revealed differences in the morphology of perovskite and carbon materials, with improved properties observed in the L6M7C3O_HT and L6M7C3O_TT samples.

Furthermore, XPS analysis revealed the significant influence of the carbon material in modifying the electronic environment of the cations, shifting the peaks towards higher binding energies. The mixing method was also found to affect the oxidation state distribution of the cations. Thus, the greater performance of the L6M7C3O_HT sample might also be related to its higher concentration of Mn^4+^ species, which benefits the interaction between active sites and oxygen species and the hydroperoxide disproportionation reaction.

## 4. Zn–Air Battery

The L6M7C3O_HT sample has been studied using a ZAB system (Figure S7). Its performance is compared to that of the L6M7C3O_BM sample, which exhibits similar ORR results. The L6M7C3O_BM sample was chosen as a reference due to its significantly simpler synthesis process, which could facilitate large-scale production.

The electrochemical performance of the synthesized materials was further analyzed using a ZAB under operating conditions.

**Figure 9:**
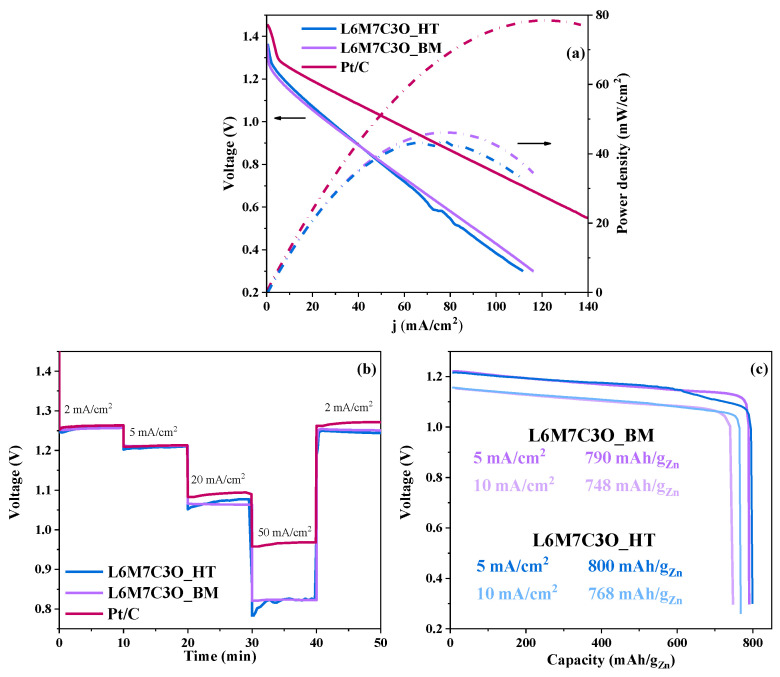
(**a**) Discharge polarization curves and power density for L6M7C3O_BM and Pt/C. (**b**) Discharge curves from 2 to 50 mA/cm^2^. (**c**) Galvanostatic discharge curves at 5 and 10 mA/cm^2^ for L6M7C3O_BM and L6M7C3O_HT samples.

In this setup, a zinc plate was used as the anode, while the air cathode (2.8 cm^2^ of geometrical area) comprised perovskite–carbon composites on carbon paper (gas diffusion layer) with a loading density of 1.3 mg/cm^2^. The electrolyte used was a mixture of 6.0 M KOH and 0.2 M Zn(O_2_CCH_3_)_2_·2H_2_O solution. A battery assembled with commercial Pt/C served as a reference for comparison. Figure 9a depicts the discharge polarization curve, where the L6M7C3O_BM sample exhibits a peak power density of 46.2 mW/cm^2^ at a current density of 78.0 mA/cm^2^ and the L6M7C30_HT sample shows a maximum power density peak at 43.9 mW/cm^2^ at 77.2 mA/cm^2^; these values do not exceed the performance of the commercial Pt/C catalyst, which achieves a maximum power of 78.4 mW/cm^2^ at a current density of 124.6 mA/cm^2^. However, differences are observed between the samples, with the L6M7C3O_BM sample exhibiting a slightly better performance at higher current densities. The sample developed in this work shows stable voltage values when subjected to different discharge current densities (2, 5, 20, 50, and back to 2 mA/cm^2^) (Figure 9b), also displaying good reversibility after operating at high current densities. Figure 9c shows the galvanostatic capacity curves at 5 and 10 mA/cm^2^ for L6M7C3O_BM (790 and 748 mAh/g_Zn_, respectively) and L6M7C3O_HT (800 and 768 mAh/g_Zn_). These values are higher than those obtained for Pt/C at 5 and 10 mA/cm^2^ (741 and 738 mAh/g_Zn_, respectively (Figure S8)).

It has been demonstrated that both samples are capable of acting as an air electrode in primary Zn–air batteries. While their performance does not surpass that of the high-activity commercial Pt/C catalyst, the focus of this work was on developing cost-effective and easily scalable electrocatalysts. Table S2 highlights the significant cost advantage of the perovskite materials used in this study (ranging from 1.55 to 25.34 USD/kg) compared to Pt (26,696.82 USD/kg), emphasizing the low-cost and simple synthesis procedures employed. Interestingly, the performance of the L6M7C3O_BM sample is very similar to that of L6M7C3O_HT. Considering its simpler synthesis process (ball milling eliminates the need for thermal treatment), we believe that the L6M7C3O_BM sample is the most promising candidate for further development.

## 5. Conclusions

It has been demonstrated that there is an effect on the electrocatalytic activity towards the ORR depending on the mixing method used between perovskite-type metal oxides and the carbon material Vulcan. The L6M7C3O_HT sample exhibited superior ORR performance compared to the others, highlighting the effectiveness of hydrothermal treatment in improving the interaction between the carbon material and the perovskite. First, XPS analysis identified a greater abundance of the M-O-C species in this material; this suggests the formation of the strongest interfacial interaction in this sample. Furthermore, TPD measurements revealed the highest desorption of oxygen for the L6M7C3O_HT sample, confirming a more intimate and extensive contact between the constituent elements. The enhancement in the interaction has led to improved electrocatalytic activity. However, electrocatalytic activity is not the only factor to consider when synthesizing electrocatalysts. This study has also highlighted the importance of cost-effective and scalable synthesis methods. The L6M7C3O_BM sample is preferable due to its simpler synthesis process (ball milling) while maintaining similar electrocatalytic activity to the L6M7C3O_HT sample. Furthermore, its performance in a primary Zn–air battery has been demonstrated. Therefore, this study proposes the use of the ball mill as a mixing method for perovskite-type metal oxides and Vulcan. Based on the findings of this work, a future line of research could be the one-step hydrothermal synthesis of perovskite–carbon composites, with the optimization of conditions such as temperature and time. This would involve synthesizing the perovskite using the hydrothermal method in the presence of the carbon material. This approach would take advantage of the improved interaction provided by the hydrothermal method, while also enhancing the simplicity of the synthesis.

## Figures and Tables

**Table 1:** Crystal phase percentages and average crystallite size for the L6M7C3O-based materials obtained from the X-ray diffraction patterns in Figure 2.

Sample	Crystallite Phases (%)		Crystallite Size (nm)		Lattice Parameter	Lattice Volume (Å^3^)
	LaMnO_3_	Mn_3_O_4_	LaMnO_3_	Mn_3_O_4_	LaMnO_3_	
L6M7C3O	88.7	11.3	18	20	3.886	58.697
L6M7C3O_BM	88.4	11.6	18	11	3.888	58.753
L6M7C3O_HT	85.3	14.7	19	15	3.888	58.771
L6M7C3O_TT	90.2	9.8	17	16	3.889	58.832

**Table 2:** Experimental atomic ratios for the materials obtained from XPS technique.

Sample	Mn^4+^/Mn^3+^	Co^3+^/Co^2+^	O_C_/O_L_	O_M-O-C_/O_L_
L6M7C3O	0.53	1.74	0.46	-
L6M7C3O_BM	0.60	1.27	0.45	0.15
L6M7C3O_HT	0.81	0.90	0.45	0.32
L6M7C3O_TT	0.76	1.00	0.31	0.15

**Table 3:** TPD quantification of the perovskite–carbon composites. Moreover, the theoretical O concentration per gram of oxide material is shown.

Sample	µmol CO/g	µmol CO_2_/g	Experimental µmol O/g	Maximum Theoretical Lattice µmol O/g
L6M7C3O	-	-	280	16,002
L6M7C3O_Mortar	1574	833	3240	
L6M7C3O_BM	1607	823	3253	
L6M7C3O_HT	1712	849	3410	
L6M7C3O_TT	1680	924	3528	
Vulcan	190	-	-	

**Table 4:** Electrochemical parameters obtained for ORR at the different samples.

Sample	$E_{onset}$/V (at −0.10 mA/cm^2^)	$n_{e^{-}}$ (at 0.4)	$j_{lim}$/mA/cm^2^ (at 0.4 V)	E_1/2_ (V)	Tafel Slope/mV/dec
L6M7C3O	0.76	3.40	−2.49	-	−169
L6M7C3O_Mortar	0.87	3.74	−4.76	0.71	−75
L6M7C3O_BM	0.88	3.76	−4.94	0.72	−63
L6M7C3O_HT	0.87	3.85	−5.36	0.75	−62
L6M7C3O_TT	0.87	3.76	−5.40	0.70	−66
Pt/Carbon	0.98	3.96	−5.51	0.86	−60
LaMn_0.7_Co_0.3_O_3__Mortar	0.84	3.78	−4.82	0.66	−81
La_0.6_MnO_z__Mortar	0.87	3.81	−4.78	0.69	−90

## Data Availability

Data are contained within the article and Supplementary Materials.

## Supplementary Materials

The following supporting information can be downloaded at https://www.mdpi.com/article/10.3390/ma17102309/s1, References [55,56,78] are cited in the supplementary materials; Figure S1: L6M7C3O perovskite TEM image; Figure S2: TEM images of the (A) L6M7C3O_Mortar, (B) L6M7C3O_BM, (C) L6M7C3O_HT and (D) L6M7C3O_TT; Figure S3: SEM images for (a) L6M7C3O_Mortar, (b) L6M7C3O_BM, (c) L6M7C3O_HT and (d) L6M7C3O_TT. Magnification: ×5000; Figure S4: Nitrogen adsorption isotherms of the composite materials; Table S1: Textural parameters obtained from the N_2_ isotherms; Figure S5: (a) RRDE linear sweep voltammograms for the comparison of the sample of this work (L6M7C3O_Mortar) with samples from previous works in 0.1 M KOH solution saturated with O_2_ at 1600 rpm, v = 5 mV/s; (b) Number of electrons transferred in ORR at increasing potential as obtained from Equation (S3) by using the current measured at the ring electrode; Figure S6: ORR Tafel slopes; Figure S7: Image of the battery system used in this work; Figure S8: Galvanostatic discharge curve at 5 and 10 mA/cm^2^ for Pt/C commercial catalysts; Table S2: Comparison of the prices of the metals used in this study and their comparison with Pt.
