# Supplementary material for: Enhancing Interaction between Lanthanum Manganese Cobalt Oxide and Carbon Black through Different Approaches for Primary Zn–Air Batteries
Source: Materials (Basel). 2024 May 13;17(10):2309. doi: 10.3390/ma17102309 (PMC11123494; doi:10.3390/ma17102309)
Supplement: Supplementary file 1 [file materials-17-02309-s001.zip › materials-2986141-supplementary.pdf]

Supporting Information

Enhancing interaction between lanthanum manganese cobalt oxide and carbon black through different approaches for primary Zn-air batteries

Mario García-Rodríguez[1], Jhony X. Flores-Lasluisa[1], Diego Cazorla-Amorós[2], Emilia Morallón[1]*

[1]Universidad de Alicante, Dept. Química Física e Instituto Universitario de Materiales, Ap. 99, E-03080, Alicante, Spain, morallon@ua.es

[2]Universidad de Alicante, Dept. Química Inorgánica e Instituto Universitario de Materiales, Ap. 99, E-03080, Alicante, Spain.

SECTION I: EXPERIMENTAL

1.1. Materials and Reagents

The reagents used were Vulcan XC-72R carbon black (Vulcan) (Cabot Corporation, Billerica, MA, USA), potassium hydroxide (KOH) (VWR Chemicals, 85%, Prague, Czech Republic), isopropanol 99.5% (Acros, Organics, 99.5%, New Jersey, USA), Nafion® 5% w/w, lanthanum(III) nitrate hexahydrate ($La(NO_3)_3\cdot 6H_2O$) (Sigma Aldrich, 99.9%, St. Louis, MO, USA), manganese(II) nitrate tetrahydrate ($Mn(NO_3)_2\cdot xH_2O$) (Alfa Aesar, 98%, Kandel, Germany), cobalt(II) nitrate hexahydrate ($Co(NO_3)_2\cdot 6H_2O$) (Sigma Aldrich, 99.9%, St. Louis, MO, USA), citric acid (Sigma-Aldrich, 99 %, St. Louis, MO, USA), ethylenediaminetetraacetic acid (EDTA) (Sigma-Aldrich, ACS reagent, St. Louis, MO, USA), ammonia (NH_3) 25 % (VWR Chemicals, analytic reagent, Billerica, MA, USA), 20% wt. Pt/C (Sigma-Aldrich, 98 %, St. Louis, MO, USA) and zinc acetate dihydrate ($Zn(O_2CCH_3)_2\cdot 2H_2O$) (Sigma Aldrich, 98 %, St. Louis, MO, USA). The solutions were prepared in ultrapure water (18 MΩ cm, Millipore® Milli-Q® water). Gases, N_2 (99.999%), O_2 (99.995%), and H_2 (99.999%) were provided by Carburos Metálicos (Spain) and used without any pretreatment.

1.1. Characterization techniques

Materials were characterized by X-ray diffraction (XRD) using a Cu a Kα radiation source at a step of 0.05° in the 2θ range from 10º to 80º on a Bruker D8-Advance

diffractometer (Billerica, USA) with a Goebel mirror (non-planar samples) and an X-ray generator KRISTALLOFLEX K 760-80F (power: 3000 W, voltage: 20–60 kV and current: 5–80 mA). The crystallite size was estimated by applying the Scherrer equation (Eq. S1) [1]:

$$D_c = \frac{k\lambda}{\beta \cos(\theta)} \tag{S1}$$

where D_c is the crystallite size (nm); k is the constant related to the shape of the grain, whose value is 0.89; λ is the wavelength of the radiation source used, which is 0.15406 nm; β corresponds to the full width at half maximum (FWHM) (radians), and θ is the Bragg angle. A crystalline quartz pattern was used to subtract the instrumental broadening factor from the measured FWHM value. Thus, the corrected FWHM value of the sample was calculated according to Eq. S2 [1]:

$$\beta_{real}^2 = \beta_{obs}^2 - \beta_{inst}^2 \tag{S2}$$

where β_{real} is the value obtained from the contribution of the crystallite size, β_{obs} is the measured value, and β_{inst} is the broadening related to the instrument.

X-ray photoelectron spectroscopy (XPS) experiments were performed in a VG-Microtech Multilab 3000 electron spectrometer (VG Scientific, Sussex, UK). The instrument was equipped with a hemispherical electron analyzer featuring nine channeltrons (passing energy of 2–200 eV). An X-ray source using Al radiation (Kα 1253.6 eV) was employed during the experiments. The C 1s peak at 284.6 eV served as an internal standard for binding energy calibration. Data analysis involved the utilization of the XPSPEAK41 program. Deconvolution of the XPS spectra was performed through the fitting of experimental curves using Lorentz-Gaussian functions, while the background subtraction was accomplished using a Shirley line.

Materials were also analyzed by temperature-programmed reduction (TPR, 5 vol % H_2 in Ar (35 mL/min), 10 °C/min up to 950 ºC) using a Micromeritics Pulse Chemisorb 2705 equipment with a thermal conductivity detector (Norcross, GA, USA).

Temperature-programmed desorption (TPD) (TA Instruments, SDT Q600 Simultaneous coupled to a mass spectrometer (PRISMA PRO QMG 250 M1)) studies were performed by heating carbon-containing samples (approximately 2 mg) from 120 to 940 °C at a heating rate of 20 °C/min under a helium flow rate of 100 mL/min. The quantity of CO

and CO_2 evolved was measured using the 28 and 44 *m*/*z* lines, respectively. These signals are calibrated employing calcium oxalate.

Sample morphologies were characterized by scanning electron microscopy (SEM, JEOL, 0.5-30kV) and by transmission electron microscopy (TEM, JEOL, 120 kV). This microscope is equipped with a field emission gun, providing high resolution (1.5 nm at 30 kV, and 4.0 nm at 1 kV), and it can operate within a voltage range from 0.5 to 30 kV.

The surface area of the samples was determined by physical adsorption-desorption of N_2 (-196 ºC) employing an automatic adsorption system (Micromeritics ASAP 2020 analyzer). Prior to the adsorption, the samples were outgassed at 250 °C under vacuum conditions for 8 h. The nitrogen adsorption data were used to calculate the Brunauer-Emmett-Teller (BET) surface area total micropore volume (Dubinin-Radushkevich equation). Moreover, the mesopore volume was calculated by the difference between the volume of N_2 adsorbed at a relative pressure of 0.95 and the micropore volume [2].

1.2. Electrochemical characterization

Electrochemical measurements were performed on an Autolab PGSTAT302 potentiostat (Metrohm, The Netherlands) with a SCANGEN module. The working electrode was a rotating ring-disk electrode (RRDE) (Pine Research Instruments, USA) with a glassy carbon disk electrode (5.00 mm in diameter) and an attached platinum ring, the counter electrode was a graphite rod, and the reference electrode was a reversible hydrogen electrode (RHE) immersed in the working electrolyte through a Luggin capillary. Before electrochemical characterization, a catalytic ink was prepared by sonicating a suspension containing 1 mg of the electrocatalysts and 1 mL of a solution composed of 0.02 vol% Nafion® and 20 vol% isopropanol in water. Then, 120 µl of the dispersion were deposited on the glassy carbon disk to generate a uniform layer of 480 µg/cm of active material. The electrochemical characterization was performed by cyclic voltammetry in either N_2- or O_2-saturated atmospheres in 0.1 M KOH at a scan rate of 50 mV/s. The electrocatalytic activity towards the ORR was studied by linear sweep voltammetry (LSV) in an O_2-saturated 0.1 M KOH solution between 1.0 and 0.0 V (vs. RHE) at different rotation rates, from 400 to 2025 rpm, and at a scan rate of 5 mV/s. Throughout all experiments, the potential of the Pt ring electrode was kept constant at 1.5 V (vs. RHE). The hydrogen peroxide formed on the disk electrode is oxidized on the Pt ring electrode and it can be

calculated according to Eq. S3. Then, the electron transfer number was determined from the current of the disk and the ring electrodes as follows (Eq. S4):

$$HO_2^-[\%] = 200x\frac{I_{ring}/N}{I_{disk}+I_{ring}/N} \quad (S3)$$

$$n_{e^-} = \frac{4I_d}{I_d+I_r/N} \quad (S4)$$

where I_r and I_d are the currents, in absolute value, measured on the ring and the disk electrodes, respectively, and N is the efficiency of ring collection, which was determined as 0.26. The ORR kinetics were evaluated using Tafel plots, $\eta = a + blog\ (j)$ where η is the overpotential and j is the current density. This analysis has been conducted within a region controlled by the electron transfer reactions and the potential range employed in each case was chosen near E_{ONSET}, not exceeding 20 mV, until achieving a linear relationship.

During Zn-air battery testing, the air cathode incorporated the most favorable samples we synthesized. These composites were loaded onto a gas diffusion layer made of carbon paper (QUINTECH, Freudenberg H23C6). The anode consisted of a polished zinc foil (ThermoScientific, 99.98%). The dimensions of the ZAB were approximately 5x5 cm and its body was made of methacrylate. In addition, silicone gaskets are used in the contact between the different components to avoid electrolyte leakage and to properly seal the system. The current collectors used were steel mesh. The catalyst ink, containing 20% isopropanol by volume and 0.02% Nafion® in water by volume, was uniformly applied to the carbon paper (surface area: 2.6 cm^2) until a mass loading of 1.3 mg/cm^2 was achieved. The electrolyte used in these experiments was a 6 M KOH and 0.2 M $Zn(O_2CCH_3)_2$ aqueous solution. A Biologic SP-300 battery testing potentiostat was employed for the tests. Polarization curves were recorded at a scan rate of 1 mA/s. For comparison purposes, the performance of a commercial Pt/C catalyst was also assessed during the tests.

SECTION II: SUPPORTING FIGURES

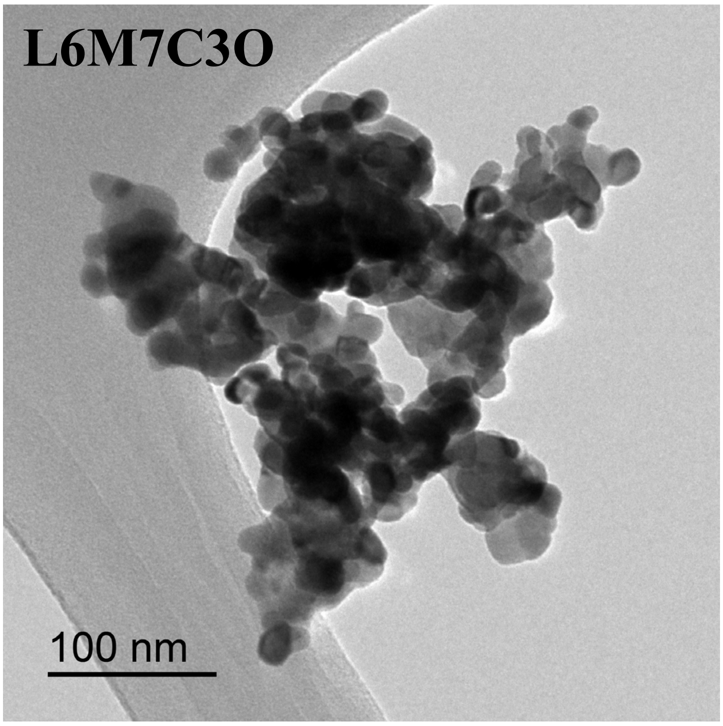

Figure S1. L6M7C3O perovskite TEM image.

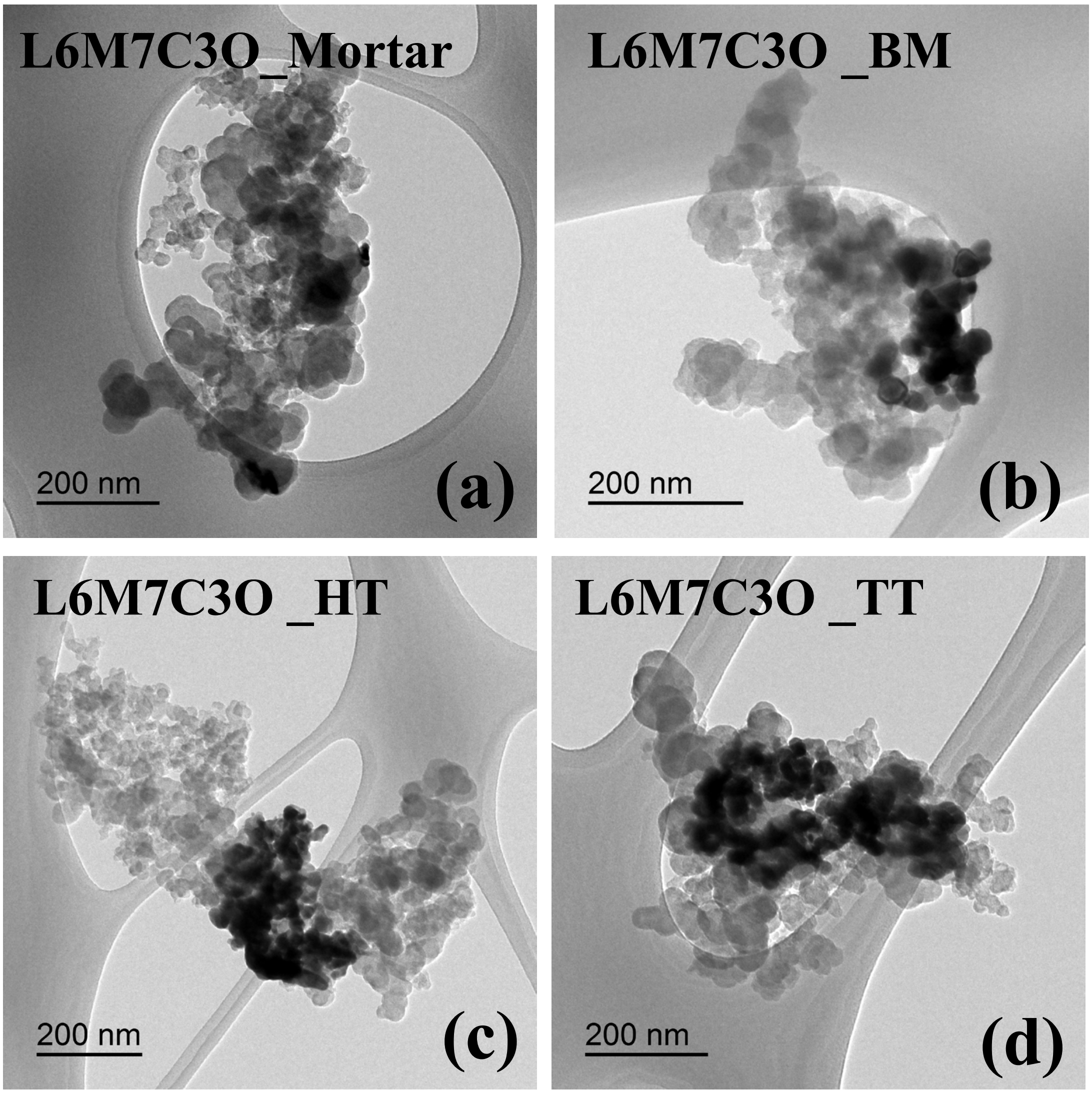

Figure S2. TEM images of the a) L6M7C3O_Mortar, b) L6M7C3O_BM, c) L6M7C3O_HT and d) L6M7C3O_TT.

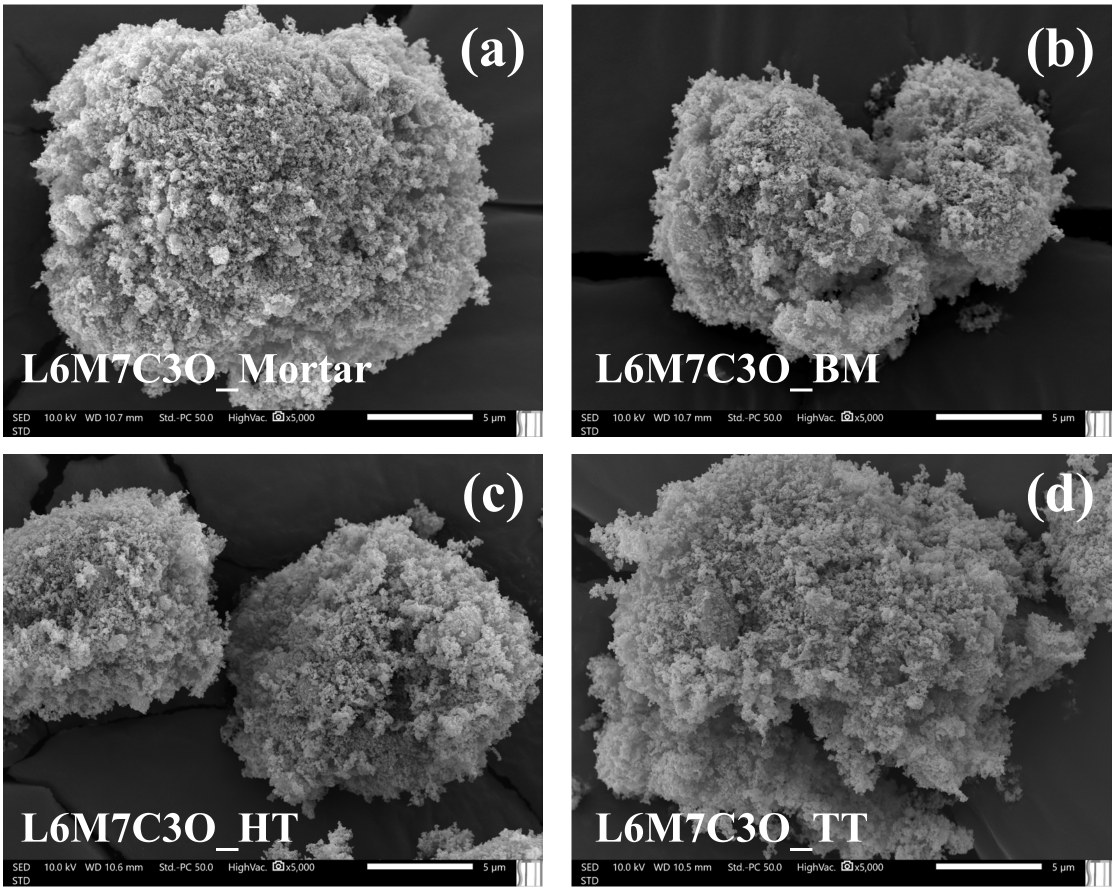

Figure S3. SEM images for a) L6M7C3O_Mortar, b) L6M7C3O_BM, c) L6M7C3O_HT and d) L6M7C3O_TT. Magnification: x5,000.

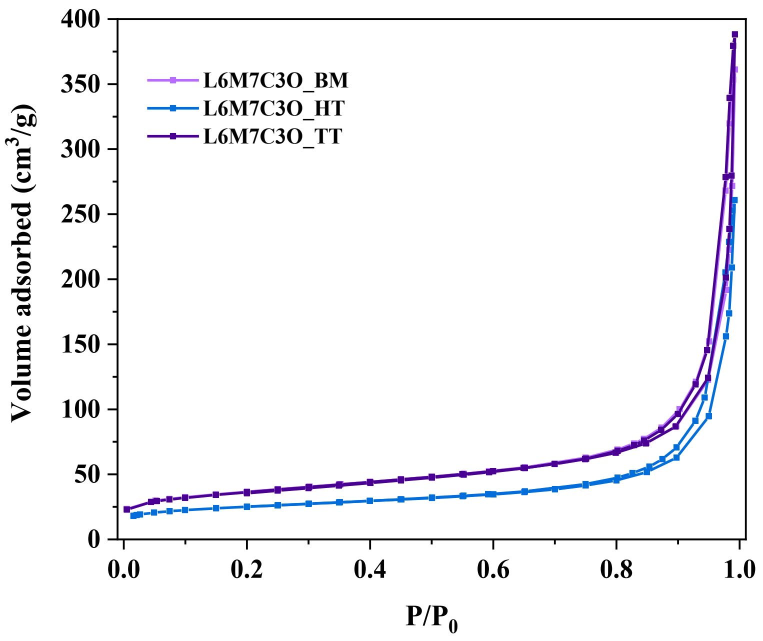

Figure S4. Nitrogen adsorption isotherms of the composite materials.

Table S1. Textural parameters obtained from the N_2 isotherms.

Sample	BET surface area/ m^2/g	Volume of micropores / cm^3/g	Volume of mesopores / cm^3/g
L6M7C3O_BM	127	0.06	0.13
L6M7C3O_HT	88	0.04	0.11
L6M7C3O_TT	126	0.06	0.14

Equation S5. Calculation of the maximum theoretical O in the L6M7C3O perovskite. It is only possible to calculate the maximum amount since oxygen vacancies are generated to compensate for the load deficiency generated by the La vacancies.

$$1g\ La_{0.6}Mn_{0.7}Co_{0.3}O_3 \times \frac{1\ mol\ La_{0.6}Mn_{0.7}Co_{0.3}O_3}{187.48\ g} \times \frac{3\ mols\ O}{1\ mol\ La_{0.6}Mn_{0.7}Co_{0.3}O_3} \times \frac{1\ \mu mol}{10^{-6}\ mol} = 16002\frac{\mu mol\ O}{g} \quad (S5)$$

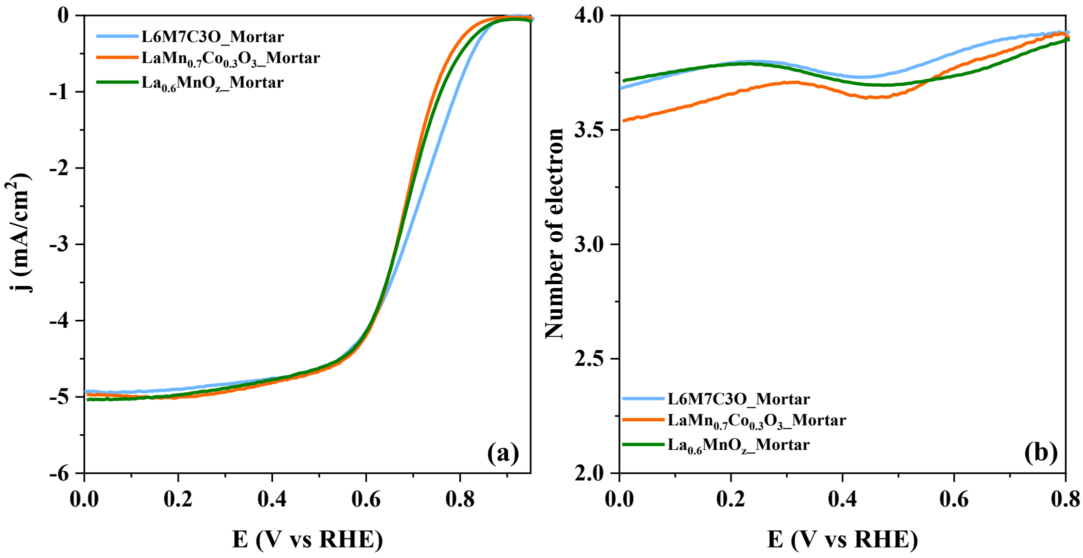

Figure S5. a) RRDE linear sweep voltammograms for the comparison of the sample of this work (L6M7C3O_Mortar) with samples from previous works in 0.1 M KOH solution saturated with O_2 at 1600 rpm, v = 5 mV/s; b) Number of electrons transferred in ORR at increasing potential as obtained from Eq. S3 by using the current measured at the ring electrode.

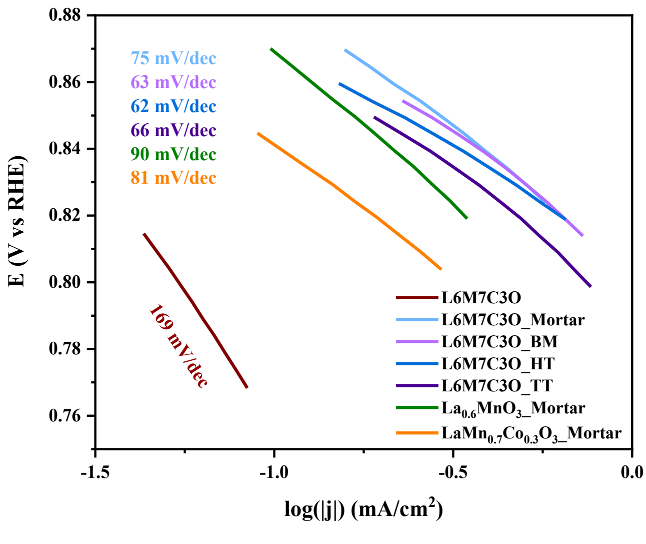

Figure S6. ORR Tafel slopes.

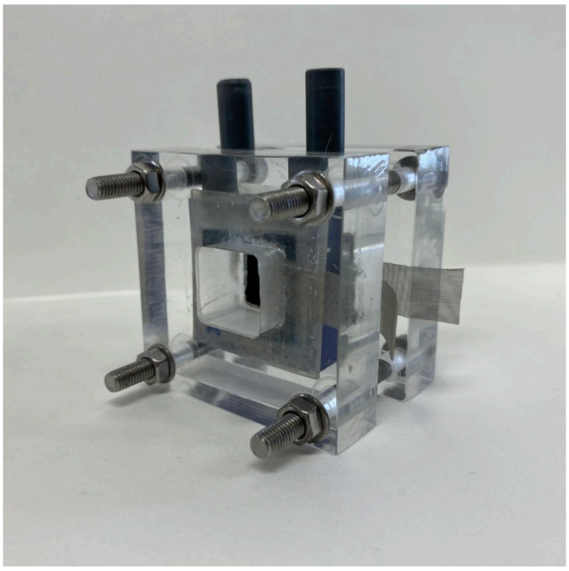

Figure S7. Image of the battery system used in this work.

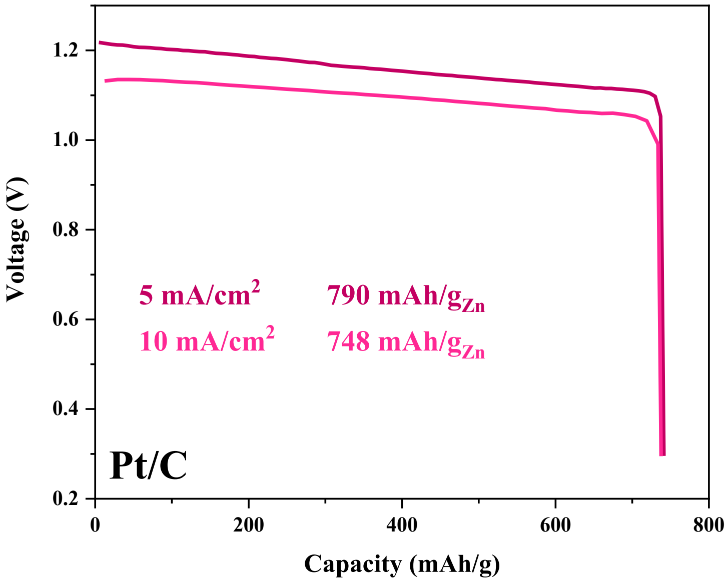

Figure S8. Galvanostatic discharge curve at 5 and 10 mA/cm^2 for Pt/C commercial catalysts.

Table S2. Comparison of the prices of the metals used in this study and their comparison with Pt [3].

Metal	USD/kg
Platinum	26696.82
Lanthanum	3.11
Electrolytic Manganese (Chongqing)	1.55
Co Powder	25.34

REFERENCES:

[1] Y. Waseda, E. Matsubara, and K. Shinoda, *X-Ray Diffraction Crystallography*. Berlin, Heidelberg: Springer Berlin Heidelberg, 2011.

[2] M. Thommes *et al.*, "Physisorption of gases, with special reference to the evaluation of surface area and pore size distribution (IUPAC Technical Report)," *Pure Appl. Chem.*, vol. 87, no. 9–10, pp. 1051–1069, 2015.

[3] "The Leading Metals Information Provider." [Online]. Available: metal.com. [Accessed: 13-Feb-2024].
